# Long-term effect of continuous positive airway pressure therapy on blood pressure in patients with obstructive sleep apnea

**DOI:** 10.1038/s41598-021-98553-0

**Published:** 2021-09-27

**Authors:** Ryutaro Shirahama, Takeshi Tanigawa, Yoshifumi Ida, Kento Fukuhisa, Rika Tanaka, Kiyohide Tomooka, Fan-Yun Lan, Ai Ikeda, Hiroo Wada, Stefanos N. Kales

**Affiliations:** 1 Department of Public Health, Graduate School of Medicine, Juntendo University, 2-1-1, Hongo, Bunkyo-ku, Tokyo, 113-8421 Japan; 2 Environmental and Occupational Medicine and Epidemiology, Environmental Health, Harvard TH Chan School of Public Health, Boston, MA USA; 3 Occupational Medicine, Cambridge Health Alliance/Harvard Medical School, Cambridge, MA USA; 4 Faculty of Science and Technology, Keio University, Kanagawa, Japan

**Keywords:** Sleep disorders, Outcomes research, Cardiac device therapy

## Abstract

Obstructive sleep apnea (OSA) is a common cause of hypertension. Previous studies have demonstrated beneficial short-term effects of continuous positive airway pressure (CPAP) therapy on blood pressure. However, long-term antihypertensive effects of CPAP have not been properly verified. This study examined the longitudinal effect of CPAP therapy adherence on blood pressure among OSA patients. All patients diagnosed with OSA and undergoing subsequent CPAP therapy at a Kanagawa-area sleep clinic were clinically followed for 24 months to examine CPAP adherence, as well as longitudinal changes in blood pressure and body weight because it may become a confound factor for changes in blood pressure. The hours of CPAP usage were collected over the course of 30 nights prior to each follow-up visit (1st, 3rd, 6th, 12th, and 24th month). The relationship between CPAP adherence and blood pressure was analyzed using mixed-effect logistic regression models. A total of 918 OSA patients were enrolled in the study. We found a significant reduction in diastolic blood pressure among patients with good CPAP adherence during the 24-month follow-up period (*β* = − 0.13, *p* = 0.03), when compared to the group with poor CPAP adherence. No significant association was found between CPAP adherence and weight loss (*β* = − 0.02, *p* = 0.59). Long-term, good CPAP therapy adherence was associated with lower diastolic blood pressure without significant weight loss**.**

## Introduction

Obstructive Sleep Apnea (OSA) is associated with hypertension^1–6^, coronary artery disease^7^, heart failure^8^, and atrial fibrillation^9^. Furthermore, nearly 50% of OSA patients have hypertension, and 37–56% of hypertensive patients have comorbid OSA^10^. The prevalence of hypertension in patients with severe OSA was 1.37 times higher than those without OSA in the Sleep Heart Health Study^11^. OSA severity is related to both nocturnal blood pressure and increases of 24-h mean blood pressure^2,12,13^.

A recent meta-analysis with 110 studies and a total of 46,188 patients found that CPAP was associated with significant changes in diurnal systolic blood pressure (weighted mean difference, − 2.4 mmHg [95% CI, − 3.9 to − 0.9]) and diurnal diastolic blood pressure (weighted mean difference, − 1.3 mmHg [95% CI, − 2.2 to − 0.4])^14^. Also, blood pressure reduction of treatment-resistant nocturnal hypertension in patients with good CPAP therapy adherence^15–17^ has been previously reported, but the observation periods of these studies were within 3 months. In another study, patients with good CPAP compliance achieved significant reductions in blood pressure at 24 months. But the subjects of the study were only 55 patients^18^. In previous study, the data was shown of the 2-year effects of CPAP treatment on blood pressure with OSA patients. The mean BMI of previous studies were over 30 kg/m^2^, the study population was different from our study^19^.

In the multicenter prospective study, the efficacy of CPAP in long-term reduction of blood pressure in patients with OSA and resistant hypertension was shown. The follow up period was 59 months and comprised of 161 patients^20^.

A previous study showed that treating OSA with CPAP may promote weight gain due to dietary intake and eating behavior^21^.

To our knowledge, no studies have examined longitudinal changes in blood pressure over multiple time points in OSA patients in relation to CPAP therapy and CPAP therapy adherence. The purpose of the present study was to investigate the effect of CPAP therapy adherence on blood pressure and body weight in a relatively lean OSA cohort with a large sample size over 2 years.

## Methods

### Subjects

This study was approved by the institutional review board of the Juntendo University, Tokyo, Japan. All patients provided written informed consent prior to participation and all subject’s information was anonymized and de-identified before analysis. The trial was performed according to the Declaration of Helsinki and Ethical Guidelines for Medical and Biological Research Involving Human Subjects of the Ministry of Education, Culture, Sports, Science and Technology. We retrospectively reviewed the medical charts of subjects with moderate to severe OSA from ages 20 to 80, who were diagnosed with OSA using overnight polysomnography (PSG) in the sleep laboratory center at RESM sleep and medical-care clinic, Kanagawa, Japan, and who started CPAP therapy between June 2013 and June 2018, were considered potentially eligible for the study. Exclusion criteria included: a lack of diagnostic polysomnography (PSG); initiation of antihypertensive agents during the study period; and failure to provide informed consent.

### Procedures

#### Initial visit

At the first visit, nurses or clinical laboratory technologists verified age and gender from the insurance card, then interviewed patients to obtain baseline demographic data, including any history of hypertension, diabetes, dyslipidemia, cerebrovascular disease, myocardial infarction, angina pectoris, smoking, alcohol intake, and the Epworth Sleepiness Scale (ESS)^22^. The average weekly alcohol intake was converted to grams of ethanol per day. Patients who smoked ≥ 1 cigarette/day were defined as current smokers, and those who had stopped smoking prior to the study were defined as former smokers. Body weight and height were measured in light clothing and without shoes on a calibrated scale and clinic stadiometer, respectively. Resting systolic and diastolic blood pressures were measured using a mercury column sphygmomanometer (No. 601, AS One, Osaka, Japan) with a cuff adapted to the arm circumference of the patient after relaxing in a chair for 10 or more minutes. The blood pressure was measured twice, and the mean systolic and diastolic values was recorded. A total of 157 subjects (131 males and 26 females) diagnosed with hypertension were treated with hypertensive drugs at the start of the study.

#### Sleep studies

All study patients underwent attended, in-laboratory, overnight PSG, Alice 6 (Philips, Amsterdam, Netherlands). PSG included continuous recording of the following: snore, chin, bilateral anterior tibialis surface electromyography, electrooculography, electroencephalography, electro cardiography, air-flow through the mouth and nose by thermistor, body posture by position sensor and SpO_2_ by oximetry. Chest and abdominal movements were recorded by respiratory inductive plethysmography^23^. The data of PSG were analyzed manually according to the American Association of Sleep Medicine (AASM) manual for the scoring of sleep and associated events^24,25^. We have clarified definitions of hypopnea and AHI and emphasized that these clarified definitions were used to analyze PSG data. A hypopnea event is defined as a reduction of air-flow by between 30 to 50% for a period of at least 10 s resulting in either a 4% reduction of oxygen saturation (SpO_2_) from baseline or an electroencephalogram arousal. AHI is defined as the total number of apnea and hypopnea events divided by the total hours of sleep^24,25^. The analysis of PSG data was done by two polysomnographic technologists certified by the Japanese Society of Sleep Research and a physician board-certified by the Japanese Society of Sleep Research.

#### CPAP therapy

All patients, between June 2013 and June 2018, diagnosed with an OSA AHI of ≥ 20 times/h were included in this study. An AHI of ≥ 20 times/h correlates to Japan insurance reimbursement requirements necessary for CPAP treatment. CPAP was titrated to the optimal pressure at which apneas and hypopneas were maximally abolished by the certified polysomnographic technologists and the certified physician using a System One/Dream Station CPAP device (Philips, Amsterdam, Netherlands). The recorded data were analyzed using a manual titration protocol.

#### Main outcome measures

Changes over time in systolic and diastolic blood pressure and body weight during CPAP therapy and relations between these parameters and adherence to CPAP therapy were assessed at multiple time points over the 24 months follow-up period. The primary outcomes were changes in systolic, diastolic blood pressure and body weight at 1st, 3rd, 6th, 12th, and 24th month.

#### Follow-up measurements

All measures were collected by certified clinical laboratory technologists. Blood pressure and weight were measured at each follow up visit in the same fashion as at baseline. Mean AHI (times/h), mean CPAP therapy use time (h/night), the rate of using CPAP therapy (% of nights) and the rate of adequate CPAP therapy use (% of nights with 4 or more hours of use in 30 days prior to each follow up visit) were analysed using data stored in the CPAP devices and cloud server via a remote monitoring system. CPAP data analysis was performed using a Dream Mapper analysis system (Philips, Amsterdam, Netherlands) or a Res Scan (ResMed, San Diego, CA, USA). The hours of CPAP usage were collected over the course of 30 nights prior to each follow-up visit (1st, 3rd, 6th, 12th, and 24th month). The rates of CPAP usage of 4 or more hours were 70% or higher was defined as good CPAP adherence, in accordance with previous studies^15,20^. Poor CPAP adherence was defined as using CPAP less time or less often than the above criteria.

### Statistical analysis

The longitudinal analyses of blood pressure and weight were performed by mixed-effect logistic regression models^26^ adjusting for age, sex, alcohol consumption, smoking, medication history of hypertension, medication history of diabetes, medication history of dyslipidemia, baseline body weight, and baseline Epworth Sleepiness Scale. Due to the analysis of repeated measures, within-individual correlation was accounted for through the use of mixed-effects regression models^27^. To examine the association of CPAP adherence to trajectories of systolic/diastolic blood pressure and body weight, we incorporated an interaction term, *good CPAP adherence* × *time*, into the models to indicate whether there were differences in the patterns of change over time in the good versus poor CPAP adherence groups.

All statistical analyses were performed using SAS version 9.2 software (SAS Institute, Cary, NC, USA) and R version 2.2.1 (R Development Core Team). Statistical significance was assessed at *p* < 0.05.

### Ethics approval

All procedures performed in studies involving human participants were in accordance with the ethical standards of the institutional and/or national research committee and with the 1964 Helsinki declaration and its later amendments or comparable ethical standards. This study protocol was approved by the institutional review board of Juntendo University, Tokyo, Japan.

### Informed consent

Informed consent was obtained from all individual participants included in the study.

## Results

### Characteristics of the subjects, diastolic sleep study and CPAP data

**Figure 1:**
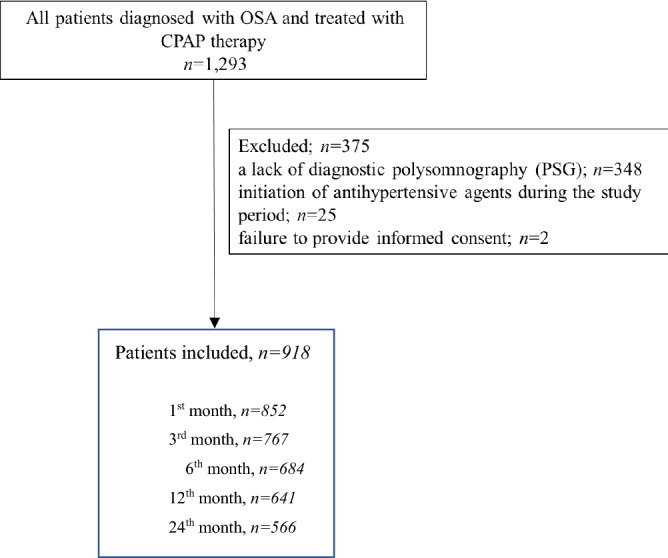
Study flow chart.

A total of 1293 subjects (1130 males; 163 females) aged 20–80 years old were diagnosed with OSA at the study clinic and received CPAP therapy during the study period. Of these, 375 patients were excluded based on the exclusion criteria, and the remaining 918 patients were enrolled in the present study (Fig. 1). Table 1 summarizes the characteristics of participants. The mean age (standard deviation) was 53.4 (12.7) years old. The mean AHI was 40.0 (19.5) times/h. The BMI was 27.0 (4.8) kg/m^2^. The baseline mean systolic and diastolic blood pressures were 133.6 (16.9) mmHg and 86.7 (12.4) mmHg. A total of 157 subjects (131 males and 26 females) diagnosed with hypertension were treated with hypertensive drugs at the start of the study. Mean residual AHI with CPAP therapy at first month was 2.9 (3.1) and that at 24 months was 2.1 (1.8) times/h. The rate of CPAP usage of 4 or more hours at 24 months was 62.3 (27.2) %.

**Table 1:** Clinical characteristics of patients and data from sleep study.

*n* = 1293	Mean (standard deviation)
Age, y	53.4 (12.7)
Body weight, kg	77.3 (16.3)
BMI, kg/m^2^	27.0 (4.8)
**Blood pressure, mmHg**	
S BP	133.6 (16.9)
D BP	86.7 (12.4)
CAVI right/left	7.45 (1.3)/7.37 (1.3)
HR, beats/minute	72.6 (16.2)
ESS	8.3 (4.5)
**Overnight polysomnography**	
AHI, events/h	40.0 (19.5)
SaO_2_ < 90, %TST	27.4 (15.5)
Mean SaO_2_, %	95.6 (2.5)
Lowest SaO_2_, %	76.1 (11.4)
**CPAP therapy**	
Residual AHI, events/h, 1 month	2.9 (3.1)
Residual AHI, events/h, 24 months	2.1 (1.8)
CPAP use ≥ 4 h/night, % nights, 24 months	62.3 (27.2)

Data expressed as mean (standard deviation); *S BP* systolic blood pressure, *D BP* diastolic blood pressure, *HR* heart rate, *CAVI* Cardio Ankle Vascular Index, *ESS* Epworth Sleepiness Scale, *AHI* apnea–hypopnea index, *TST* total sleep time, *BMI* body mass index, *SaO*_*2*_ arterial oxyhemoglobin saturation.

### Changes in blood pressure and body weight after CPAP therapy

#### The whole sample of patients, good vs poor adherers to CPAP, address all time points

**Figure 2:**
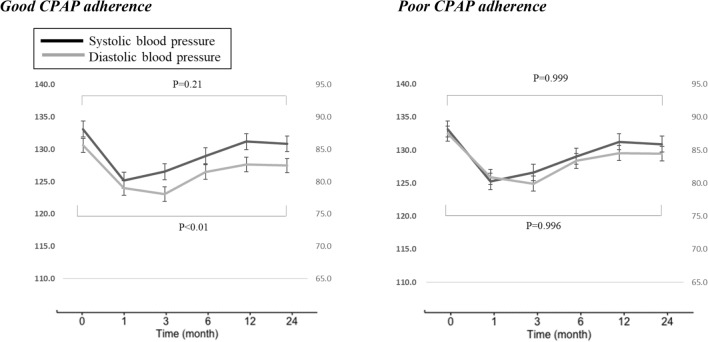
Correlation between changes in systolic and diastolic blood pressure and adherence of continuous positive airway pressure. Thick line (left axis), systolic blood pressure (mmHg); Thin line (right axis), diastolic blood pressure (mmHg); Good CPAP adherence, time using CPAP more than 4 h ≥ 70% of the 30 days prior to all follow-up points; Poor CPAP adherence, time using CPAP more than 4 h ≤ 70% of the 30 days prior to all follow-up points. Time, month of CPAP treatment. Upper P values are related to systolic blood pressure and lower p values are related to diastolic blood pressure. Model has adjusted for age and body weight.

In the multivariate analyses, systolic and diastolic blood pressures decreased significantly in both good and poor adherence groups from baseline to the first month after the initiation of CPAP. However, systolic and diastolic blood pressures increased from month 1 to month 12 and decreased from month 13 thru month 24. Moreover, a significant diastolic blood pressure reduction was found in patients with good CPAP adherence (Fig. 2). Body weight tended to increase over the 24-month observation period in both the good and poor CPAP adherence groups from 77.3 (± 16.3) kg to 78.3 (± 16.0) kg. Though this was the case we did not find a significant longitudinal association between time × good adherence and body weight.

#### The whole sample of patients, good vs poor adherers to CPAP, address baseline and final assessment

**Figure 3:**
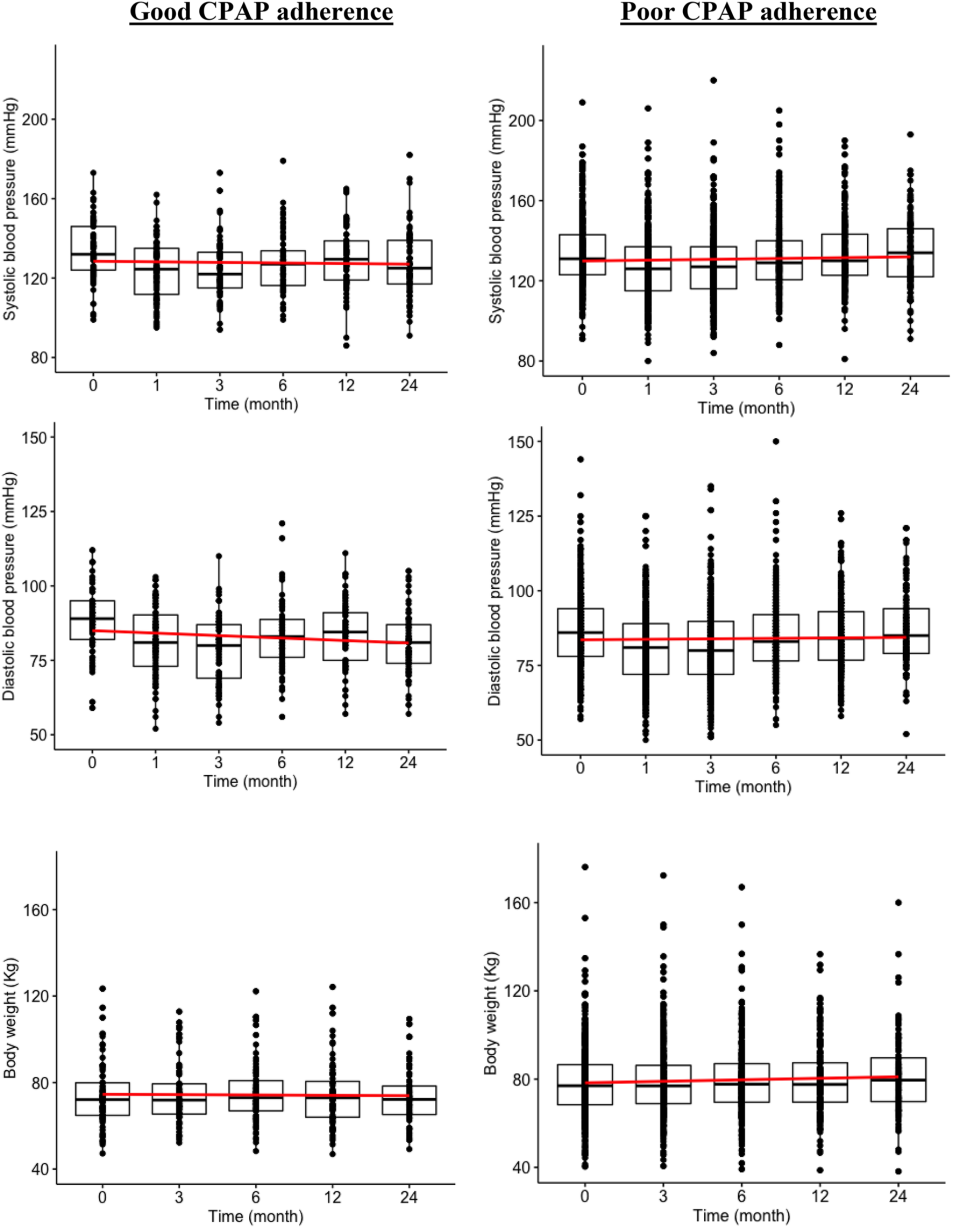
Association between longitudinal changes in blood pressure and body weight and adherence of continuous positive airway pressure. The red lines are temporal trends of mean blood pressure derived using linear regression functions. Good CPAP adherence, using CPAP for more than 4 h ≥ 70% of the time of the 30 days prior to all follow-up points.; Poor CPAP adherence, using CPAP for more than 4 h ≤ 70% of the time of the 30 days prior to all follow-up points. Time, month of CPAP treatment. Model was adjusting for age, sex, alcohol consumption, smoking, medication history of hypertension, medication history of diabetes, medication history of dyslipidemia, body weight, and Epworth Sleepiness Scale.

To examine the association of CPAP adherence to trajectories of systolic/diastolic blood pressure and body weight, we incorporated an interaction term, good CPAP adherence × time, into the models to indicate whether there were differences in the patterns of change over time in the good versus poor CPAP adherence groups. The results based on mixed-effect logistic regression models showed significant reduction of diastolic blood pressure trajectories comparing good to poor CPAP adherence patients during 24-month follow-up period, after multivariate adjustment. We found a significant longitudinal association between time × good CPAP adherence and diastolic blood pressure (β = − 0.13, p = 0.03). The coefficient estimate of β = − 0.13 indicates that blood pressure decreased by (an average of) 0.13 mmHg every month (Fig. 3; Table 2). We did not find a significant longitudinal association between time × good CPAP adherence and systolic blood pressure (*β* = − 0.14, *p* = 0.12) or CPAP adherence and body weight (*β* = − 0.02, *p* = 0.59).

**Table 2:** Multivariate-adjusted *β* estimates for blood pressure and body weight associated with CPAP adherence.

	Systolic blood pressure		Diastolic blood pressure		Body weight	
	*β* (S.E.)	*P*	*β* (S.E.)	*P*	*β* (S.E.)	*p*
Time (2-year)	0.21 (0.05)	< 0.001	0.11 (0.03)	0.002	0.02 (0.02)	0.33
Good CPAP adherence	− 0.8 8 (1.71)	0.60	1.00 (1.36)	0.46	− 0.24 (1.88)	0.90
Time × Good CPAP adherence	− 0.14 (0.09)	0.12	− 0.13 (0.06)	0.03	− 0.02 (0.04)	0.59

*S.E.* standard error; Good CPAP adherence, using CPAP for more than 4 h ≥ 70% of the 30 days prior to all follow-up points. Model was adjusting for age, sex, alcohol consumption, smoking, medication history of hypertension, medication history of diabetes, medication history of dyslipidemia, baseline body weight, and baseline Epworth Sleepiness Scale.

#### The whole sample of patients, good vs poor adherers to CPAP, normotensive vs hypertensive subjects, address baseline and final assessment

We also found a significant longitudinal association between time × high CPAP adherence and systolic blood pressure (β = − 0.20, p < 0.05) or time × high CPAP adherence and diastolic blood pressure stratified for normotensive patients (β = − 0.18, p = 0.01). We did not find a significant longitudinal association between time × good CPAP adherence and body weight stratified for normotensive (β = − 0.02, p = 0.67) and hypertensive patients (β = − 0.01, p = 0.81) (Table 3).

**Table 3:** Multivariate-adjusted β estimates for blood pressure and body weight associated with CPAP adherence stratified for normotensive and hypertensive patients.

	Systolic blood pressure		Diastolic blood pressure		Body weight	
	*β* (S.E.)	*P*	*β* (S.E.)	*P*	*β* (S.E.)	*p*
**Hypertensive patients (n = 157)**						
Time (2-year)	0.19 (0.10)	0.10	0.09 (0.07)	0.19	0.0 (0.03)	0.87
Good CPAP adherence	− 4.49 (3.54)	0.20	− 0.80 (2.54)	0.75	1.8 (4.0)	0.63
Time × Good CPAP adherence	0.05 (0.20)	0.78	0.02 (0.13)	0.86	− 0.01 (0.06)	0.81
**Normotensive patients (*n* = 409)**						
Time (2-year)	0.22 (0.06)	< 0.001	0.11 (0.04)	0.005	0.03 (0.02)	0.28
Good CPAP adherence	0.21 (1.93)	0.91	1.27 (1.58)	0.42	− 1.63 (2.14)	0.48
Time × Good CPAP adherence	− 0.20 (0.10)	< 0.05	− 0.18 (0.07)	0.01	− 0.02 (0.05)	0.67

*S.E.* Standard Error; Good CPAP adherence, using CPAP for more than 4 h ≥ 70% of the 30 days prior to all follow-up points. Model was adjusting for age, sex, alcohol consumption, smoking, medication history of hypertension, medication history of diabetes, medication history of dyslipidemia, baseline body weight, and baseline Epworth Sleepiness Scale.

## Discussion

In a large cohort of OSA patients, we found a significant difference in diastolic blood pressure trajectories when comparing patients with good or poor CPAP adherence during the 24-month follow-up period, after multivariate adjustment. The patient group with good CPAP adherence (a mean usage rate of CPAP for more than 4 h ≥ 70% calculated from each point) showed a significant diastolic blood pressure reduction during the follow-up period, indicating a significant longitudinal effect of CPAP therapy. Stratified for normotensive and hypertensive patients, it seems good CPAP adherence was beneficial for normotensive patients (p < 0.05), while the results were not significant in hypertensive patients. Small sample size after stratification is a limitation, and may reduce the statistical power to detect differences in hypertensive patients.

Blood pressure reduction of treatment-resistant nocturnal hypertension in patients with good CPAP therapy adherence^15–17^ has been previously reported, but the observation periods of these studies were only 2–3 months. In the previous study, patients with good CPAP compliance achieved significant reductions in blood pressure at 24 months. But the subjects of the study were only 55 patients^18^. In the recent study, the efficacy of CPAP in long-term reduction of blood pressure in patients with OSA and resistant hypertension was shown. The follow up period was 59 months and comprised of 161 patients. In a previous study the 2-year effects of CPAP treatment on blood pressure with OSA patients was shown^19^. The mean BMI of this previous study were over 30 kg/m^2^. Compared to this study the mean BMI of our study was 27 kg/m^2^. To the best of our knowledge, this is the first study specifically designed to investigate the long-term effects of CPAP treatment on blood pressure in a relatively lean OSA-diagnosed patient population- a mean BMI of less than 30 kg/m^2^.

The World Health Organization (WHO) defines a BMI of 30 kg/m^2^ or higher as "obese". Obese populations may have a more pronounced BP-lowering response to good CPAP compliance than relatively lean populations because obese populations are in a state of sympathetic hypertonia due to excessive insulin secretion. Body weight tended to increase over the 24-month observation period in both the good and poor CPAP adherence groups. However, we do not believe that weight changes, taken at timepoints of our study, had a material influence on BP of either good or poor CPAP adherence patient population**.**

This suggests that CPAP therapy directly contributes to blood pressure reduction, without mediation by weight loss. Further, we performed multiple imputation to impute missing data, which did not alter the association between diastolic blood pressure and CPAP adherence.

Our results showed long term (24 months) use of CPAP in both good and poor adherence CPAP users reduced both systolic and diastolic blood pressure. In the first month after the initiation of CPAP systolic and diastolic blood pressures decreased from baseline significantly in both good and poor adherence groups. However, systolic and diastolic blood pressures varied from that point- increasing from month 1 to month 12, then decreasing from month 13 thru month 24. Importantly, a significant diastolic blood pressure reduction was found in patients with good CPAP adherence (Fig. 2). We estimated the changes due in part to (1) balance between the effect of suppressing sympathetic over strain and (2) arteriosclerosis advancing with aging—a primary reason for the increase of blood pressure. These variations of systolic and diastolic blood pressure would not have been able to be seen without repeated assessments over time.

*We speculate that the reduction of blood pressure at the end of month 1 of CPAP therapy was associated with acute improvements in functional vascular damage. Improvements in endothelial function and sympathetic overactivation by short-term CPAP therapy have been reported^28,29^. The increase in blood pressure from months 2 thru 12 months was due to natural progression associated with the aging process. Ultimately, the decrease in blood pressure from months 13 thru 24 seemed to be due to chronic improvements in endothelial and autonomic functions. Additional research is planned to further examine the long-term effect of CPAP on arterial stiffness.

Good CPAP adherence for normotensive patients seemed beneficial, while the results for hypertensive patients were not significant. Hypertensive patients may have a more pronounced BP-lowering response to good CPAP adherence than normotensive subjects. Improvements in normotensive patients were all the more impressive because most hypertensive patients were on β blockers, which could in and of itself counteract the surges or activity within the sympathetic nervous system that blunt baroreflex sensitivity. Good CPAP adherence has the potential to reset the baroreflex threshold downward. This change could not only reset BP to a lower level but also improve its regulation. There are also several other potential mechanisms to reduce blood pressure associated with CPAP therapy. CPAP holds the airway open, preventing collapse and improving ventilation, which suppresses vascular endothelial dysfunction by stabilizing oxygenation and reduces oxidative stress^30^. Furthermore, CPAP therapy has been correlated with a down-regulation of the renin–angiotensin system activity^31^, with a reduction of plasma aldosterone^32^. Also, by reducing sleep disruption, CPAP suppresses sympathetic nervous activity^33^.

In the present study, patients with good CPAP adherences demonstrated a significant reduction in diastolic blood pressure when compared to the group with poor CPAP adherence. However, no significant associations were found between systolic blood pressure and CPAP adherence. Previous studies showed a correlation of CPAP adherence with 24-h diastolic pressure reduction, however, not with 24-h systolic blood pressure reduction^34,35^. CPAP therapy was more effective in treatment-resistant hypertension patients than in treatment-responsive hypertension patients, suggesting that CPAP therapy improved excess sympathetic nerve tone and altered vascular responses^36^. Decrease in blood pressure by CPAP therapy especially for diastolic pressure are believed to be due to decreased peripheral vascular resistance^30^.

The major strength of our study is its longitudinal design. We recorded both blood pressure and body weight at multiple time points over two years. Also, we had a larger cohort of OSA patients compared to previous studies, which enabled us to further stratify the analysis by potential confounders and covariates.

There are several limitations to the present study. First, our study had the inherent weakness of a retrospective design, which may bias the results. However, it was longitudinal and observational as opposed to random assignment to good or poor adherence. And we compared the difference between good and poor CPAP adherence groups, without an untreated group as a control. Antihypertensive medication started or changed during the follow-up, the patients were excluded from study. Therefore, the antihypertensive effect of CPAP therapy shown in this study may be underestimated, not overestimated. Second, we used office blood pressure which is less accurate to assess blood pressure than 24 h ambulatory blood pressure monitoring. However, the blood pressure was measured twice after relaxing in a chair for 10 or more minutes using a mercury column sphygmomanometer, and the mean systolic and diastolic values was recorded. Third, the good CPAP adherence group is likely to also be more adherent to a healthier lifestyle such as balanced diet and regular exercise. And potential life habits that may influence blood pressure (e.g., salt intake, physical activity) were not considered in the study. Unfortunately, these lifestyle data were not available.

In conclusion, the patient group with good CPAP adherence, and no weight loss over two years, showed a significant reduction of diastolic blood pressure indicating a significant longitudinal effect of CPAP therapy on diastolic blood pressure reduction. Therefore, a significant potential reduction in cardiovascular morbidity and mortality may be expected in normotensive OSA patients. However, more research is recommended to further clarify the clinical relevance of our findings.

## References

[1] Peppard PE, Young T, Palta M (2000). Prospective study of the association between sleep-disordered breathing and hypertension. N. Engl. J. Med..

[2] Nagata K, Osada N, Shimazaki M (2008). Diurnal blood pressure variation in patients with sleep apnea syndrome. Hypertens. Res..

[3] Pedrosa RP, Drager LF, Gonzaga CC (2011). Obstructive sleep apnea: The most common secondary cause of hypertension associated with resistant hypertension. Hypertension.

[4] Kario K (2009). Obstructive sleep apnea syndrome and hypertension: Mechanism of the linkage and 24-h blood pressure control. Hypertens. Res..

[5] Dudenbostel T, Calhoun DA (2012). Resistant hypertension, obstructive sleep apnoea and aldosterone. J. Hum. Hypertens..

[6] Pekar Y, Hedner J, Norum J (2002). Increased incidence of cardiovascular disease in middle-aged men with obstructive sleep apnea: A 7-year follow-up. Am. J. Respir. Crit. Care Med..

[7] Kent BD, Garvey JF, Ryan S (2013). Severity of obstructive sleep apnoea predicts coronary artery plaque burden: A coronary CT angiography study. Eur. Respir. J..

[8] Kato M, Adachi T, Koshino Y (2009). Obstructive sleep apnea and cardiovascular disease. Circ. J..

[9] Latina JM, Estes NA, Garlitski AC (2013). The relationship between obstructive sleep apnea and atrial fibrillation: A complex interplay. Pulm. Med..

[10] Drager LF, Genta PR, Pedrosa RP (2010). Characteristics and predictors of obstructive sleep apnea in patients with systemic hypertension. Am. J. Cardiol..

[11] Nieto FJ, Young TB, Lind BK (2000). Association of sleep-disordered breathing, sleep apnea and hypertension in a large community-based study, Sleep Heart Health Study. JAMA.

[12] Wolk R, Shamsuzzaman AS, Somers VK (2003). Obesity, sleep apnea, and hypertension. Hypertension.

[13] Pickering TG, Kario K (2001). Nocturnal non-dipping: What does it augur?. Curr. Opin. Nephrol. Hypertens..

[14] Jonas DE, Amick HR, Feltner C (2017). Screening for obstructive sleep apnea in adults: Evidence report and systematic review for the US Preventive Services Task Force. JAMA.

[15] Martínez-García MA, Capote F, Campos-Rodríguez F (2013). Effect of CPAP on blood pressure in patients with obstructive sleep apnea and resistant hypertension: The HIPARCO randomized clinical trial. JAMA.

[16] Weaver TE, Grunstein RR (2008). Adherence to continuous positive airway pressure therapy: The challenge to effective treatment. Proc. Am. Thorac. Soc..

[17] Martínez-García MA, Gómez-Aldaraví R, Soler-Cataluña JJ (2007). Positive effect of CPAP therapy on the control of difficult-to-treat hypertension. Eur. Respir. J..

[18] Barbé F, Durán-Cantolla J, Sánchez-de-la-Torre M (2012). Effect of continuous positive airway pressure on the incidence of hypertension and cardiovascular events in nonsleepy patients with obstructive sleep apnea. JAMA.

[19] Bouloukaki I, Mermigkis C, Tzanakis N (2017). The role of compliance with PAP use on blood pressure in patients with obstructive sleep apnea: Is longer use a key-factor?. J. Hum. Hypertens..

[20] Navarro-Soriano C, Torres G, Barbé F (2020). The HIPARCO-2 study: Long-term effect of continuous positive airway pressure on blood pressure in patients with resistant hypertension: A multicenter prospective study. J. Hypertens..

[21] Tachikawa R, Ikeda K, Minami T (2016). Changes in energy metabolism after continuous positive airway pressure for obstructive sleep apnea. Am. J. Respir. Crit. Care Med..

[22] Johns MW (1991). A new method for measuring daytime sleepiness: The Epworth sleepiness scale. Sleep.

[23] Shirahama R, Wada H, Tanigawa T (2017). Current medical treatment of obstructive sleep apnea (OSA) in sleep center. Juntendo Med J..

[24] Berry RB, Brooks R, Vaughn BV (2017). AASM Scoring Manual Updates for 2017 (Version 2.4). J. Clin. Sleep Med..

[25] Berry RB, Budhiraja R, Tangredi MM, American Academy of Sleep Medicine (2012). Rules for scoring respiratory events in sleep: Update of the 2007 AASM Manual for the Scoring of Sleep and Associated Events. Deliberations of the Sleep Apnea Definitions Task Force of the American Academy of Sleep Medicine. J. Clin. Sleep Med..

[26] Hunger M, Doring A, Holle R (2012). Longitudinal β regression models for analyzing health-related quality of life scores over time. BMC Med. Res. Methodol..

[27] Hu FB, Goldberg J, Hedeker D, Flay BR (1998). Comparison of population-averaged and subject-specific approaches for analyzing repeated binary outcomes. Am. J. Epidemiol..

[28] Ip MS, Tse HF, Lam B (2004). Endothelial function in obstructive sleep apnea and response to treatment. Am. J. Respir. Crit. Care Med..

[29] Narkiewicz K, Kato M, Phillips BG, Pesek CA (1999). Nocturnal continuous positive airway pressure decreases daytime sympathetic traffic in obstructive sleep apnea. Circulation.

[30] Montesi SB, Edwards BA, Malhotra A (2012). The effect of continuous positive airway pressure treatment on blood pressure: A systematic review and meta-analysis of randomized controlled trials. J. Clin. Sleep Med..

[31] Campos-Rodriguez F, Perez-Ronchel J, Grilo-Reina A (2007). Long-term effect of continuous positive airway pressure on BP in patients with hypertension and sleep apnea. Chest.

[32] Kohler M, Craig S, Pepperell JCT, Stradling JR (2013). CPAP improves endothelial function in patients with minimally symptomatic OSA: Results from a subset study of the MOSAIC trial. Chest.

[33] Nicholl DD, Hanly PJ, Poulin MJ (2014). Evaluation of continuous positive airway pressure therapy on renin–angiotensin system activity in obstructive sleep apnea. Am. J. Respir. Crit. Care Med..

[34] Lloberes P, Sampol G, Espinel E, Tovar JL (2014). A randomized controlled study of CPAP effect on plasma aldosterone concentration in patients with resistant hypertension and obstructive sleep apnea. J. Hypertens..

[35] Logan AG, Tkacova R, Perlikowski SM (2003). Refractory hypertension and sleep apnoea: Effect of CPAP on blood pressure and baroreflex. Eur. Respir. J..

[36] Hu X, Fan J, Chen S (2015). The role of continuous positive airway pressure in blood pressure control for patients with obstructive sleep apnea and hypertension: A meta-analysis of randomized controlled trials. J. Clin. Hypertens..

